# Novel genetic polymorphisms associated with severe malaria and under selective pressure in North-eastern Tanzania

**DOI:** 10.1371/journal.pgen.1007172

**Published:** 2018-01-30

**Authors:** Matt Ravenhall, Susana Campino, Nuno Sepúlveda, Alphaxard Manjurano, Behzad Nadjm, George Mtove, Hannah Wangai, Caroline Maxwell, Raimos Olomi, Hugh Reyburn, Christopher J. Drakeley, Eleanor M. Riley, Taane G. Clark, in collaboration with MalariaGEN

**Affiliations:** 1 Pathogen Molecular Biology Department, London School of Hygiene and Tropical Medicine, London, United Kingdom; 2 Department of Immunology and Infection, London School of Hygiene and Tropical Medicine, London, United Kingdom; 3 Centre for Statistics and Applications, University of Lisbon, Lisbon, Portugal; 4 Joint Malaria Programme, Kilimanjaro Christian Medical College, Moshi, Tanzania; 5 National Institute for Medical Research, Mwanza, Tanzania; 6 Department of Infectious Disease Epidemiology, Faculty of Epidemiology and Population Health, London School of Hygiene and Tropical Medicine, London, United Kingdom; Case Western Reserve University School of Medicine, UNITED STATES

## Abstract

Significant selection pressure has been exerted on the genomes of human populations exposed to *Plasmodium falciparum* infection, resulting in the acquisition of mechanisms of resistance against severe malarial disease. Many host genetic factors, including sickle cell trait, have been associated with reduced risk of developing severe malaria, but do not account for all of the observed phenotypic variation. Identification of novel inherited risk factors relies upon high-resolution genome-wide association studies (GWAS). We present findings of a GWAS of severe malaria performed in a Tanzanian population (n = 914, 15.2 million SNPs). Beyond the expected association with the sickle cell HbS variant, we identify protective associations within two interleukin receptors *(IL-23R* and *IL-12RBR2)* and the kelch-like protein *KLHL3* (all P<10^−6^), as well as near significant effects for Major Histocompatibility Complex (MHC) haplotypes. Complementary analyses, based on detecting extended haplotype homozygosity, identified *SYNJ2BP*, *GCLC* and MHC as potential loci under recent positive selection. Through whole genome sequencing of an independent Tanzanian cohort (parent-child trios n = 247), we confirm the allele frequencies of common polymorphisms underlying associations and selection, as well as the presence of multiple structural variants that could be in linkage with these SNPs. Imputation of structural variants in a region encompassing the glycophorin genes on chromosome 4, led to the characterisation of more than 50 rare variants, and individually no strong evidence of associations with severe malaria in our primary dataset (P>0.3). Our approach demonstrates the potential of a joint genotyping-sequencing strategy to identify as-yet unknown susceptibility loci in an African population with well-characterised malaria phenotypes. The regions encompassing these loci are potential targets for the design of much needed interventions for preventing or treating malarial disease.

## Author summary

Malaria, caused by *Plasmodium falciparum* parasites, is a major cause of mortality and morbidity in endemic countries of sub-Saharan Africa, including Tanzania. Some gene mutations in the human genome, including sickle cell trait, have been associated with reduced risk of developing severe malaria, and have increased in frequency through natural selection over generations. However, new genetic mutations remain to be discovered, and recent advances in human genome research technologies such as genome-wide association studies (GWAS) and fine-scale molecular genotyping tools, are facilitating their identification. Here, we present findings of a GWAS of severe malaria performed in a well characterised Tanzanian population (n = 914). We confirm the expected association with the sickle cell trait, but also identify new gene targets in immunological pathways, some under natural selection. Our approach demonstrates the potential of using GWAS to identify as-yet unknown susceptibility genes in endemic populations with well-characterised malaria phenotypes. The genetic mutations are likely to form potential targets for the design of much needed interventions for preventing or treating malarial disease.

## Introduction

Sub-Saharan Africa bears a disproportionately high share of the global *Plasmodium falciparum* malaria burden, with 90% of the estimated 212 million annual cases and 92% of 429,000 annual deaths, mostly in children under five years of age **[1].** Whilst the majority of cases of *Plasmodium falciparum* infection are asymptomatic or cause only mild to moderate clinical symptoms, a subset of affected individuals present with severe manifestations such as severe malarial anaemia and cerebral malaria. Risk factors for severe malaria and its various clinical subtypes are poorly understood, although host and parasite genotype, age and immune status have all been established as playing a significant role in individual host susceptibility **[2]**. *Plasmodium falciparum* has also exerted significant selection pressure upon the human genome, as evidenced by the geographical concurrence of malaria parasite prevalence with sickle cell trait (HbAS) and other haemoglobinopathies, such as the thalassemias and glucose-6-phosphate dehydrogenase (G6PD) deficiency.

Recent studies, set in a region of high malaria transmission in north-eastern Tanzania, estimated that host genetic factors account for approximately 22% of the total variation in severe malaria risk **[3]**, consistent with previous findings in a Kenyan family-based study **[2].** Less than half of this variation can be explained by erythrocyte-associated polymorphisms **[4]**, including *HbS* (sickle cell trait), alpha-thalassaemia, ABO blood group **[5]** and G6PD deficiency **[4]**. Novel polymorphisms in or around *USP38*, *FREM3* **[3],** glycophorins *gypA/B/E* **[6, 7],** *DDC* **[8]**, *MARVELD3* and *ATP2B4* **[9]** account for additional variation but, in sum, are less protective than heterozygous carriage of *HbS* **[3].** Moreover, the effects of some of these loci are subtype-, location-, or population-specific **[3, 6, 7, 9]**, reinforcing the need for targeted genome-wide association studies (GWAS) in different African populations. Utilising such an approach with robust malaria phenotypes in parallel with whole genome sequencing of study populations is crucial to unravelling host genetic factors that could lead to a greater understanding of protective immunity and development of new tools for disease prevention.

To identify novel loci associated with severe malaria in north-eastern Tanzania, we applied genome-wide association and haplotype-based selection methods to a case-control dataset with extensive phenotypic data for 914 participants and 15.2 million SNPs. In addition to the expected *HbS* (sickle cell) association, our analyses reveal multiple novel loci under association or selection. Association analysis highlighted significant SNPs clusters within *IL-23R*, *IL-12RB2*, *LINC00944*, and *KLHL3* whilst lone SNP associations were also present within *TREML4* and *ZNF536*. Further, we reveal loci under recent positive selection including *GCLC* and loci within the Major Histocompatibility Complex (MHC). These analyses were supported by whole genome sequencing of an independent dataset consisting of 247 Tanzanian individuals within parent-child trios, which was used to confirm the allele frequencies of putative associations and determine if there are any linked common structural variants in chromosome regions encoding important polymorphisms.

## Results

### Phenotypic and genotypic data

All severe malaria cases (n = 449) and controls (n = 465) came from the Tanga region of North-Eastern Tanzania. Severe malaria cases presented with varying combinations of hyperlactataemia (57.0%), severe malarial anaemia (49.2%), respiratory distress (27.6%) and cerebral malaria (26.7%) (**Table 1**). Compared to controls, malaria cases were younger (t test P<2.2_x10_^-16^) and marginally more likely to be male (Chi squared P = 0.012) **(Table 1)**. DNA from all samples (n = 914) was genotyped on the Illumina Omni 2.5 million SNP chip, and imputed against the 1000 Genomes reference panel (Phase 3) **[10]** and a Tanzanian parent-child trio panel (see below), using Beagle 4.1 **[11],** leading to 15.2 high quality SNPs. These markers were complemented by 180 SNPs within malaria candidate genes, including *HBB* (encoding HbS) **[3, 4, 5]** on the same cases and controls. DNA from a validation cohort of 78 healthy parent and child trios and 13 independent individuals (“Trios dataset”, n = 247) were whole genome sequenced using Illumina HiSeq2500 technology. For the GWAS samples, a principal component analysis (PCA) using all genome-wide SNPs revealed a low degree of stratification by ethnicity and case-control status (**S1 Fig**) and potential cryptic relatedness due to familial clustering. A similar analysis revealed that GWAS and Trio sample clusters overlap, and there is some separation from the other 1000 Genome African populations, including Yoruba (Nigeria) and Luhya (Kenya) (**S1 Fig**).

**Table 1:** Study participants.

	Controls (n = 465)		Cases (n = 449)		Difference P-value
Age* (median, range)	2.8	0.9–10.9	1.7	0.2–10.0	<2.2_x10_^-16^
Female	252	54.2%	205	45.7%	0.012
Ethnicity**					0.52
Mzigua	151	32.5%	146	32.5%	
Wasambaa	142	30.5%	135	30.1%	
Wabondei	83	17.8%	86	19.2%	
Mmbena	26	5.6%	23	5.1%	
Mngoni	17	3.7%	18	4.0%	
Pare	11	2.4%	8	1.8%	
Mmakonde	11	2.4%	8	1.8%	
Mgogo	7	1.5%	8	1.8%	
Chagga	9	1.9%	7	1.6%	
Other	8	1.7%	10	2.2%	
Mixed Ethnicity***	150	32.3%	172	38.2%	0.065
Hyperlactatemia/acidosis	-	-	256	57.0%	-
Severe Malarial Anaemia	-	-	221	49.2%	-
Respiratory Distress	-	-	124	27.6%	-
Cerebral Malaria	-	-	120	26.7%	-

* months

** based on paternal ethnicity

*** if parental ethnicities were different

### Association analysis

**Fig 1:**
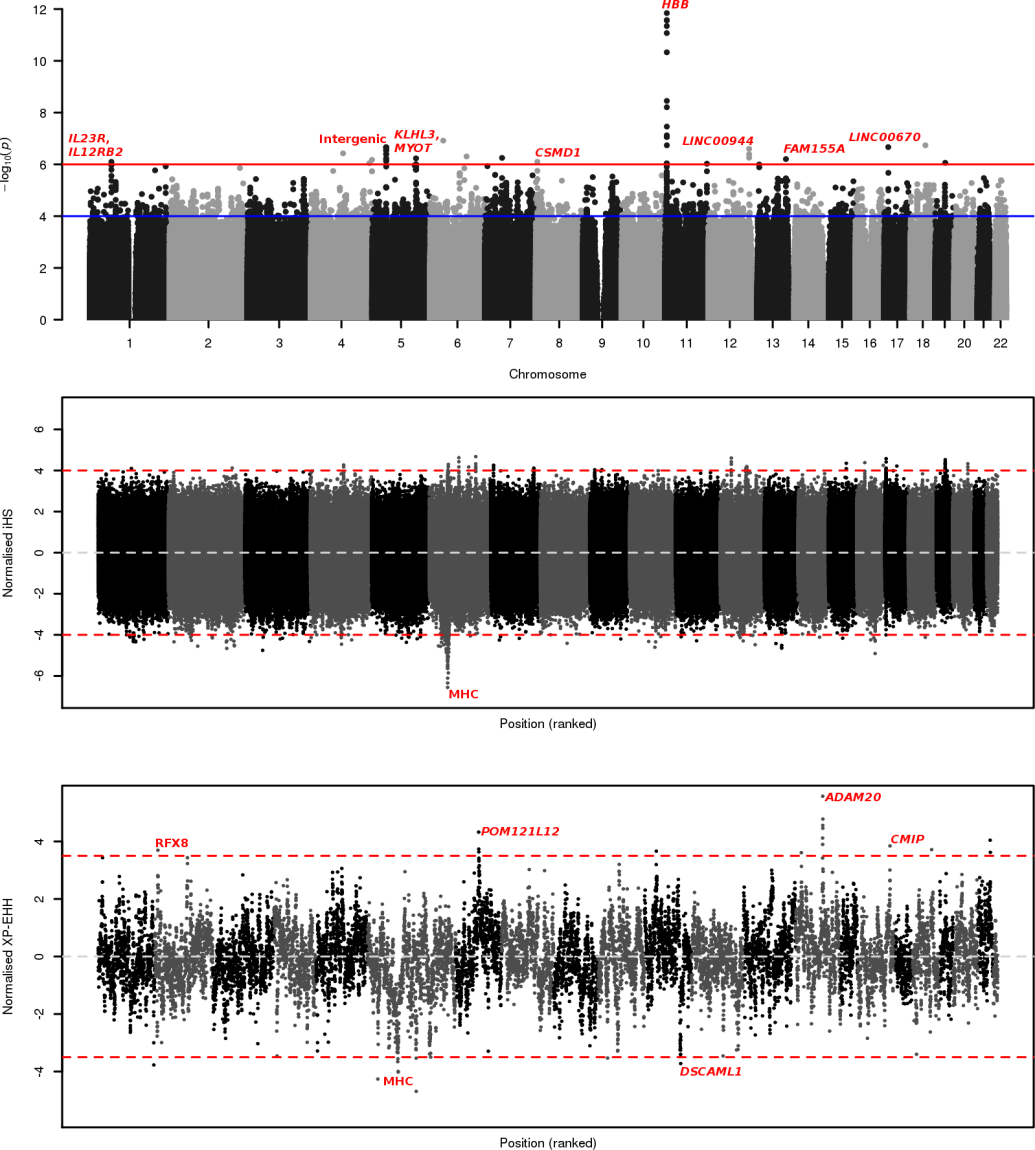
Genome-wide association and selection plots. a) Case-Control SNP Associations. Red line indicates genome-wide significance at 1_x10_^-6^, blue line indicates genome-wide suggestive significant at 1_x10_^-4^; b) Combined population iHS selection. Red lines indicate significance for absolute iHS scores of 4 or greater; c) Case-Control XP-EHH selection. Red lines indicate significance for absolute XP-EHH scores of 4 or greater.

GWAS analysis was undertaken with EMMAX mixed model regression **[12]**, controlling for age as a fixed effect and relatedness (represented in a kinship matrix) as a random effect to account for the cryptic population clustering. Separate models of association were fitted for each SNP (additive, heterozygous, dominant, recessive), with their respective genomic inflation factors all being close to one (see **S1 Fig** for the heterozygous results), consistent with reliable adjustment for stratification. A total of 53 SNPs (in 16 genomic regions) were identified with a significance level below our threshold (P<1_x10_^-6^) **(Fig 1, Table 2, S1 Table**). Relaxing the stringency would lead to 258 SNPs with a p-value below 1_x10_^-5^ and 2,322 below a threshold of 1_x10_^-4^. As expected, the most significant association was with the sickle cell locus, *rs334* (P = 8.59_x10_^-13^, heterozygous odds ratio = 0.07) (**Table 2**). Controlling for HbS status through a complementary conditional GWAS demonstrated our top associations as robust against linkage with *rs334* (**Table 2, S1 Table)**.

**Table 2:** More significant SNP associations per locus (P<1x10-6).

SNP	Gene	n SNPs	Location	Minimum P	Conditional P	Subtype P
rs334	HbS (in *HBB*)	40	11:5248232	HET: 8.59E-13	-	HL: 1.81e-09
rs9296359	*TREML4*	1	6:41205690	HET: 1.21E-07	HET: 4.42e-07	SMA: 3.29e-07
rs149085856	Intergenic (LINC00670)	1	17:12399526	ADD: 2.15E-07	ADD: 1.06e-06	HL: 2.81e-07
rs113449872	Intergenic	20	5:43909343	HET: 2.17E-07	HET: 2.93e-07	SMA: 2.92e-05
rs11335470	*LINC00944*	3	12:127237620	HET: 2.52E-07	HET: 1.86e-06	HL: 9.04e-05
rs73832816	*C4orf17*	1	4:100429757	REC: 3.75E-07	REC: 9.48e-07	CM: 1.02e-06
rs17624383	Intergenic	3	7:53676837	ADD: 5.62E-07	ADD: 3.28e-06	RD: 4.61e-07
rs2967790	*KLHL3*, *MYOT*	13	5:137011761	ADD: 5.85E-07	ADD: 2.46e-06	HL: 8.65e-06
rs144312179	*FAM155A*	6	13:108228013	ADD: 6.24E-07	ADD: 2.92e-06	HL: 1.35e-06
rs114169033	AF146191.4–004 (lincRNA)	3	4:190717704	ADD: 6.67E-07	ADD: 1.30e-06	RD: 5.62e-07
rs6682413	*IL23R*, *IL12RB2*	7	1:67731614	REC: 7.98E-07	REC: 1.03e-06	SMA: 1.23e-04
rs73505850	*CSMD1*	5	8:4754838	ADD: 7.98E-07	ADD: 1.42e-06	SMA: 1.20e-05
rs8109875	*ZNF536*	1	19:31069639	REC: 8.69E-07	REC: 3.57e-06	SMA: 2.80e-05
rs1878468	AC108142.1 (antisense)	1	4:182822332	HET: 8.98E-07	HET: 1.19e-06	HL: 8.10e-07
rs3133394	Intergenic	4	11:130417522	ADD: 9.41E-07	ADD: 1.08e-06	CM: 9.49e-06

Allele models: ADD Additive, HET Heterozygous, DOM Dominant, REC Recessive. Subtype significances: HL Hyperlactatemia; SMA Severe Malarial Anaemia; RD Respiratory Distress; CM Cerebral Malaria. Locations correspond to the GRCh37 reference genome. Minimum P indicates the most significant P for SNPs in the locus within the case-control GWAS, whilst Conditional and Subtype Ps indicate the most significant P value for those SNPs when controlling for rs334 status, or considering the severe malarial subtypes.

**Fig 2:**
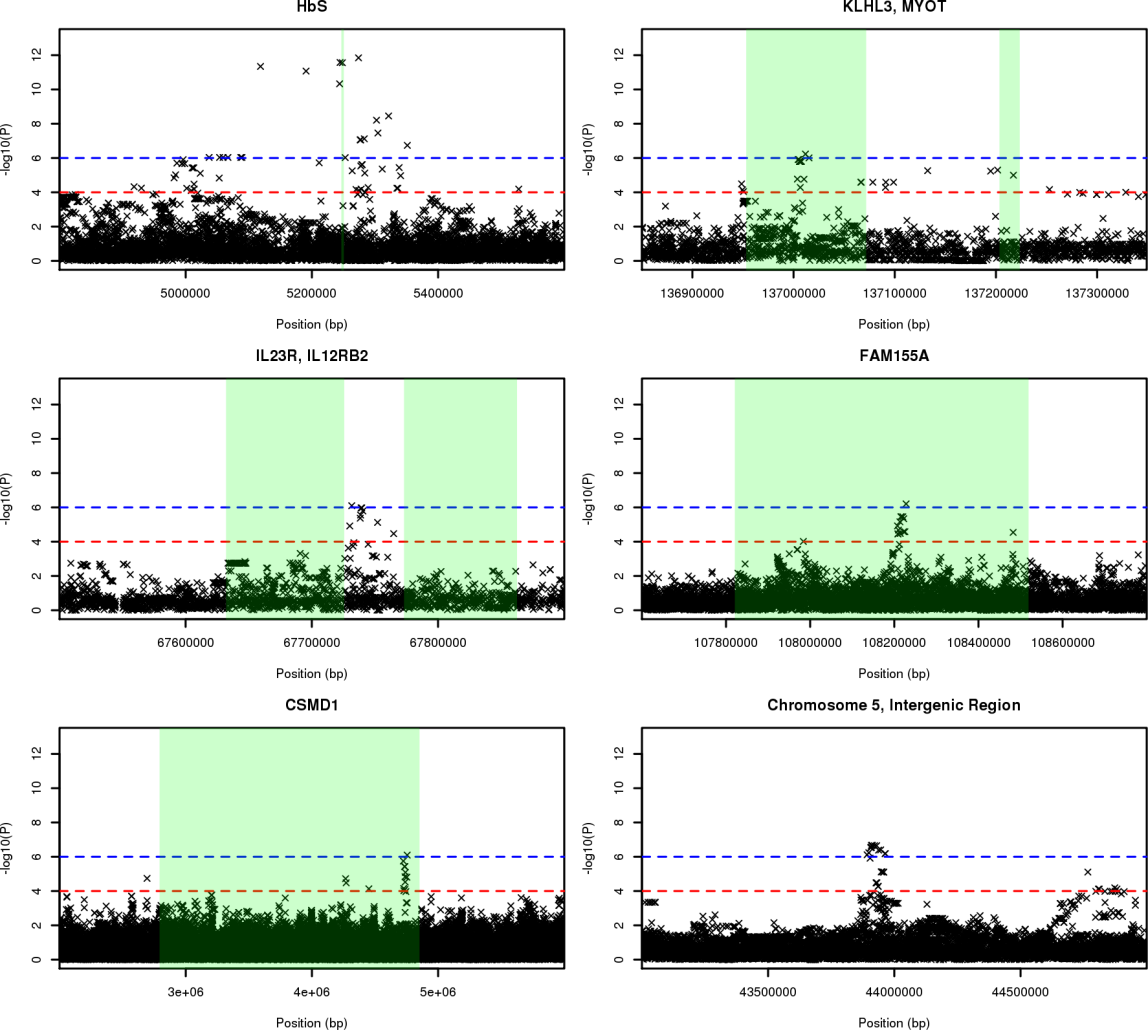
Region plots for most significant SNP associations. Green regions highlight genes of interest, as indicated by sub-plot headers. The red line indicates genome-wide significance at 1_x10_^-6^, whilst the blue line indicates genome-wide suggestive significant at 1_x10_^-4^.

Novel associations of note also include SNPs within the *KLHL3-MYOT* region (13 SNPs, Min P = 5.85_x10_^-7^, Additive OR = 0.590), the *IL23R-IL12RB2* region (7 SNPs, Min P = 7.98_x10_^-7^, Recessive OR = 0.479), *FAM155A* (6 SNPs, Min P = 6.24_x10_^-7^, Additive OR = 0.207), and *CSMD1* (5 SNPs, Min P = 7.98_x10_^-7^, Additive OR = 4.795). (**Fig 2**). Three significant SNPs are also found within both *LINC00943/4* and *lincRNA AF146191*.*4–004*.

Lone SNP associations are present within proximity of *TREML4* (P = 1.21_x10_^-7^, Heterozygous OR = 4.087), zinc finger-containing *ZNF536* (P = 8.69_x10_^-7^, Recessive OR = 0.507), *C4orf17* (P = 3.75_x10_^-7^, Recessive OR = 0.289), and near *LINC00670* (P = 2.15_x10_^-7^, Additive OR = 3.867). And finally, three intergenic regions display clusters of significance, most notably a region within chromosome 5 (43,892,232–43,964,366bp; Min P = 2.17_x10_^-7^, Heterozygous OR = 0.354), as well as regions within chromosome 7 and 11.

As expected, allele frequencies of the putative polymorphisms within the Trios dataset are generally equivalent to frequencies in our case and control groups, whilst there were some differences from the 1000 Genomes populations, including within the *HBB* locus (**Table 2**). Using the Trios dataset, we sought to identify structural variants that could confound the association analysis or be putative hits. We identified no structural variants within *HBB*, *IL-12RBR2* or LINC00943/4, one deletion (2,904bp) within *IL23R*, and 152 deletions within *KLHL3* (63 distinct variants, all singletons except for one 1,325bp deletion in 91 individuals) **(S2 Table)**. None of the common variants are in linkage disequilibrium with the putative GWAS hits, and eight putative regions had structural variants in the Tanzanian trios, but were absent in the 1000 Genomes populations **(S2 Table).**

Subtype specific association analyses were undertaken for those SNPs found to be significantly associated with severe malaria in the primary GWAS (**Table 2**). The majority of significant associations are with the hyperlactataemia subtype, a phenotype that includes 57.0% of cases, with variants within *FAM155A*, and the *HBB* and *KLHL3*/*MYOT* regions exhibiting associations exceeding our 1_x10_^-6^ significance threshold. In contrast, variants within *IL-23R*, *IL-12RB2*, *CSMD1*, *ZNF536* and *TREML4* appear to be most significantly associated with severe malarial anaemia, who comprised 49.2% of cases.

### Candidate associations

Candidate SNPs identified in previous studies, with the same individuals, were included to provide appropriate context for novel findings. ABO blood group, *USP38*, *FREM3* and alpha-thalassemia have previously been putatively associated with severe malaria in a Tanzanian population **[3, 5]**, but these associations are no longer statistically significant (P>10^−4^) at a more stringent GWAS significance threshold (**S3 Table**). We also performed targeted imputation of HLA haplotypes within the MHC, finding the most significant SNP to be rs1264362, which demonstrated a marginal association with hyperlactatemia (additive model P = 2.33_x10_^-5^).

For the analysis of structural variation within candidate regions in the Trios dataset, we identified 28 distinct deletions within *FREM3*, of which all but one are present in only one individual, and six distinct deletions in *GYPB*, for which copy number variation has previously been identified **[6].** Nine distinct variants were identified in *ABO*, including six duplications, one deletion, one insertion and one inversion. All such *ABO* variants are present in single individuals, though 18 individuals have a 23bp insertion. In contrast to a diversity of structural variation present within HLA and the wider MHC region, minor frequency variants were identified in *ATP2B4* (25 deletions across 25 samples), *MARVELD3* (five deletions across five samples), *HBA2* (3 deletions across three samples), and *HBA1* (one sample with one deletion). No structural variants were found in *HBB* or *USP38* (**S2 Table)**.

We imputed structural variants within the wider region of human glycophorin genes (*gypA*, *gypB*, *gypE*) on chromosome four, using 55 distinct large polymorphisms identified in 59 individuals within our Trios dataset (**S2 Table**). The glycophorin region is structurally highly diverse, and specific individual variants are of low frequency (mean frequency: Case Control dataset = 0.098, Trios dataset = 0.022), consistent with observations in other African populations **[7].** Whilst these large variants could be potentially protective against severe malaria, we identified no significant associations looking at each individually (P ≥ 0.301). Grouping these variants into forms based on genomic location and function may enhance signals within this region, but could also introduce experimenter bias. Further, there exists a multitude of potential variant combinations analysis of which, without specific hypotheses, could risk so-called ‘P hacking’. A full and in-depth analysis of this region is required but beyond the scope of this study.

### Evidence of positive selection

Two approaches were applied to identify regions under recent positive selection within the Tanzanian GWAS population as a whole (Integrated Haplotype Score, iHS) **[13]**, or between the cases and controls (Cross-Population Extended Haplotype Homozygosity, XP-EHH) **[14].** A common genome-wide absolute score threshold of 4 (equivalent to P = 6.3_x10_^-5^) was established for both approaches. At this threshold iHS identified 244 loci, 116 (47.5%) within chromosome 6. Ninety-four of these significant signals are within the MHC, with three loci within *HLA-DOA* having an absolute score greater than 6. Other MHC genes with significant signals include the immunophilin *FKBP5* (35732-37931bp, 9 SNPs, iHS: 4.00–4.84), *SAMD3* (12490-13053bp, 6 SNPs, iHS: 4.00–4.68) and the exocytosis regulator *RIMS1* (72805811-72828559bp, 3 SNPs, iHS: 4.17–4.62) (**S4 Table**). Most notably, two regions within chromosome 17 (3496105-3689132bp, 6 SNPs, 8 genes including integrin *ITGAE*) and chromosome 19 (38743962-38900106bp, 14 SNPs, 10 genes including two transmembrane channels) represent regions with a high density of selection signals, akin to those within the MHC. Further signals of note include the transcription factor *ZFHX3* (*ATBF1*) (chr. 16, 16326-73133bp, 3 SNPs, iHS: 4.27–4.91), *ABHD5* (chr. 3, 43794949bp, 1 SNP, iHS: 4.75), *DUSP19* & *NUP35* (chr. 2, 99180–18528, 3 SNPs, iHS: 4.30–4.66), surface tyrosine-kinase receptor *ERBB4* (chr. 2: iHS: 4.31–4.54), transcription-associated *RORC* (chr. 1, 151792842-151817543bp, 2 SNPs, iHS: 4.31–4.66).

No structural variation was identified in *ABHD5* or *DUSP19*, whilst variants were present but rare for the remaining iHS hits **(S2 Table).** In total, two deletions were identified in *RORC*, three in *ZFHX3*, *NUP35*, and *ITGAE*, one of which is an 86bp deletion found in seven individuals, and 14 deletions and a 31bp insertion in *RIMS1*. Particularly variable are *ERBB4* and *FKBP5* for which we identify 49 and 75 distinct variants respectively. *ERBB4* consists of 44 deletions, four insertions, and one inversion, whilst *FKBP5* consists of 70 deletions, three duplications, one insertion, and one inversion.

The between case-control XP-EHH approach identifies 10 significant SNPs across six genetic regions (**S5 Table**). Relative selection for the control population lies within three regions, including *POM121L12* (XP-EHH: 4.33), *SYNJ2BP*, *ADAM21*, *ADAM20* (XP-EHH: 4.12 to 5.57) and *ERG* (XP-EHH: 4.04), whilst three regions are under relative selection in the case population, including *MCUR1* (XP-EHH: -4.26), *GCLC* (XP-EHH: -4.69) and the MHC (XP-EHH: -4.02) (**S5 Table**). We identify no structural variants within *POM121L12*, *ADAM21*, or *ADAM20*, but a singleton 75bp deletion in *SYNJ2BP*, two deletions within *MCUR1*, three deletions in *GCLC*, and 20 distinct deletions within *ERG*, of which 8 individuals share a 106bp deletion and 6 share a 325bp deletion **(S2 Table).**

## Discussion

As expected, the most significant SNP association is the heterozygous protective *rs334* effect (P = 2.61_x10_^-13^), with thirty-nine further SNPs within *HBB* also being significantly associated with resistance to severe disease. SNPs in other candidate genes, including *FREM3*, *GYPA*, *GYPB* and *USP38* **[3],** did not exceed a significance threshold of 1_x10_^-6^, and their p-values were different (greater) to those previously published because of the use of the more conservative EMMAX mixed model regression **[12].** Marginal evidence for a role of HLA association with severe malaria was also identified, and is broadly consistent with previous work in a West African population that demonstrated that carriers of HLA Class I Bw53 and HLA class II DRB1*1302-DQB1*0501 were protected against severe malaria **[15].** Note that our targeted imputation of HLA utilised a Caucasian reference panel and may therefore overlook further true associations within the HLA locus. Further, we identified signals of positive selection within the MHC region, this being consistent with malaria as a driver of MHC polymorphism in the human population **[16, 17].**

Of the novel SNP associations identified here, two of the top candidates are located between the interleukin receptors *IL-23R* and *IL-12RB2*, a region that has been identified in GWAS of other inflammatory and immune-linked diseases **[18].** *IL-12* and *IL-23* are related pro-inflammatory cytokines that share both the p40 subunit and the *IL-12Rβ1* receptor subunit. *IL-12* signals through a receptor comprising *IL-12Rβ1* and *IL-12Rβ2* and is a potent inducer of *IFN-γ* which mediates both clearance of infection and immunopathology in infections with Plasmodium parasites. *IL-23* signalling (through its receptor, comprising *IL-12Rβ1* and *IL-23R*) promotes transcription of *RORC* which encodes *RORγ*, a transcription factor involved in generation of *IL-17*. *RORC* was found to be under recent positive selection in our analysis, further supporting the importance of the pathway. Decreased *IL-12* levels have been associated with progression from uncomplicated malaria to severe disease, specifically an increased risk of severe malarial anaemia in children **[19, 20].** Variants in *IL-12B* have been linked to *P*. *falciparum* parasite density and associated with protection against cerebral malaria in children whilst, variants in the related *IL-12A* and *IL-12RB1* loci have been associated with protection against severe malarial anaemia among children in western Kenya **[19].** Conversely, the *IL-23*/*IL-17* immune pathway has been implicated in the development of inflammatory reactions in children that develop severe malarial anaemia **[21],** in multi-organ dysfunction and acute renal failure in adult *P*. *falciparum* cases from India **[22]** and with the risk of cerebral malaria in Africa **[23].** *IL-23R* haplotypes have also been associated with increased susceptibility to severe malarial anaemia in Kenya **[24].**

Three significantly associated SNPs are present within *LINC00944*, with one being 80bp from a known CTCF binding site **[25].** Structurally, although the LINC00943/4 region is a known deletion site **[25],** we identified no such deletions within the region in our ‘Trios’ dataset. Broader functionality of this long intergenic non-coding RNA is unclear, given limited experimental characterisation, making it difficult to determine a role for these SNP variants.

A strong association peak was also identified within *KLHL3*, kelch-like protein 3, being a region known to contain an enhancer and various deletions **[25].** Correspondingly, we identify 152 such deletions within our Trios reference panel, of which 62 distinct variants are present in only one individual and one 1,325bp deletion is present in 91 individuals. This frequent deletion is located within an open chromatin-containing region between 137,022,562 and 137,023,887bp. Mutations of *KLHL3* have previously been linked with hypertension and metabolic acidosis **[26]** suggesting that these novel SNP associations and deletions may prime individuals to have a greater risk for severe malarial acidosis (hyperlactataemia).

A number of the most significantly associated SNPs are present as lone, or paired, associations rather than “stacks”. This includes SNPs within or very near to *TREML4* and *ZNF536*. Whilst this may demonstrate false positive outliers, the existence of these SNPs and their minor frequencies are confirmed in our Trios reference panel.

The broad picture of whole population iHS selection is unsurprising, with the MHC region demonstrating the most striking evidence for recent selective sweeps. Our results are also consistent with a number of previously identified iHS signals, such as those for loci containing the alcohol dehydrogenase *ADH7*, cadherin *PCDH15*, synaptotagmin *SYT1*, the nociception receptor *TRPV1*, and the transmembrane protein *SPINT2* **[27].** It should also be emphasised that our iHS signals reflect selection within our case-control dataset and therefore oversample, relative to a general Tanzanian population, for those signals associated with susceptibility to severe malaria.

Recent differential selection between the case and control groups, as determined by XP-EHH, identified very few significant signals. There is likely to be limited differential selection between subsets of a closely related population, despite malaria infection being a strong selector. We identified the MHC, *GCLC*, *MCUR1*, *POM121L12* and the *SYNJ2BP*-*ADAM21*-*ADAM20* region. The strongest of these signals covers *ADAM20* and *ADAM21*, both members of a larger family of disintegrins and metalloproteinases that are believed to be exclusively expressed in the testis **[28];** this association might simply reflect differences in the gender ratio between the cases and controls, for which XP-EHH does not control. Selection for this region is more likely driven by a variant of *SYNJ2BP*, a Synaptojanin-2 binding protein with potential roles in membrane trafficking and signalling **[29]**.

Our previous work has demonstrated that novel associations with potentially significant roles in malaria susceptibility remain to be uncovered **[3]**, and here we show that an integrated approach that identifies signals of association, selection and structural variation can empower such studies. However, with only 914 individuals in this study, sample size is a notable limitation for interpretation. Initial approaches to account for this were pursued through robust contextualisation of novel variants within the secondary ‘Trios’ dataset, and the wider 1000 Genomes project. More generally, it remains vital that further validation, through larger scale studies, be undertaken to better characterise the SNP and structural variants uncovered. This is particularly true for structural variation such as within *KLHL3*, which may impact gene expression and would therefore benefit from incorporation of transcriptomic data.

Distributions of human genetic variants with putative roles in *P*. *falciparum* malaria susceptibility are diverse. The HbS sickle cell polymorphism is present across most regions of sub-Saharan Africa but is known to have arisen multiple times leading to a number of distinct haplotypic backgrounds **[30].** Similarly, other variants, such as *G6PD* polymorphism and glycophorin structural variants vary both in frequency across populations and in their direction of association, leading in some cases to allelic heterogeneity that may be subtype specific. Many protective variants identified within our study, such as *IL-23R* and *KLHL3*, were found at similar frequencies within the ‘Trios’ dataset but differed from the global 1000 Genomes panel, and may therefore represent examples of Tanzanian- or regional-specific associations. Such variants are informative to our understanding of human-parasite interactions, yet risk being overlooked in inadequately designed studies. Ultimately, human GWAS in parallel with whole genome sequencing of host and parasites in large study populations across Africa will be crucial to unravelling host genetic and parasite interactions that could lead to novel malaria control measures such as vaccines.

## Methods

### Ethics statement

All DNA samples were collected and genotyped following signed and informed written consent from a parent or guardian. Ethics approval for all procedures was obtained from both LSHTM (#2087) and the Tanzanian National Institute of Medical Research (NIMR/HQ/R.8a/Vol.IX/392).

### Study participants and phenotypes

All participants were from the Tanga region of North-Eastern Tanzania, as described previously **[3]**. Briefly, severe malaria cases (n = 449) were recruited in the Teule district hospital and surrounding villages in Muheza district, Tanga region, Tanzania between June 2006 and May 2007. The controls (n = 465) were recruited, matched on ward of residence, ethnicity and age (given in months), during August 2008 from individuals without a recorded history of severe malaria **[3]**. Four severe malaria subtypes were identified within case individuals including hyperlactatemia (Blood lactate > 5 mmol/L, n = 256), severe malarial anaemia (Hemocue Hb < 5g/dL, n = 221), respiratory distress (n = 124) and cerebral malaria (Blantyre coma score <5, n = 120) (**Table 1**). Parasite infection was initially assessed by rapid diagnostic test (HRP-2 –Parascreen Pan/Pf) and confirmed by double read Geimsa-stained thick blood films.

A further 247 anonymously sampled individuals, consisting of 78 healthy parent and child trios (156 parents, 78 children, 13 singletons; 80 (32.4%) Chagga, 77 (31.2%) Pare, 90 (36.4%) Wasambaa), were collected between 2007 and 2008. These individuals are those that had no current illness or no history of malaria. The samples were collected from highland, medium and lowland villages near the Kilimanjaro, Pare and West Usambara mountains in the Tanga region of Tanzania. This is a region that experiences low to medium to high levels of malaria transmission. This dataset was used to confirm allele frequencies and identify candidate region structural variation within the general Tanzanian population, as well as to impute variants onto the case-control set.

### Sample genotyping, sequencing and imputation

DNA was extracted from processed blood samples, as described previously **[3, 5]**. The DNA was genotyped on the Illumina Omni 2.5 million SNP chip and SNP genotypes called by the MalariaGEN Resource Centre at the Sanger Institute and the Wellcome Trust Centre for Human Genetics, using previously described methods **[6,7]**. These data were complemented by Iplex genotyping assays that included 180 single nucleotide polymorphisms (SNP) across 50 loci on the same individuals **[3].** 107 additional candidate SNPs, including the HbS SNP *rs334*, were included from previous candidate genotyping of the same case-control individuals; their collection having been described previously **[3].** DNA for the individuals in the Trio dataset (n = 247) was sequenced using Illumina HiSeq2500 technology at the Sanger Institute, and aligned to the GRCh37 build of the human genome **[7]**. The minimum genome-wide coverage across the samples was 22-fold. SNPs were called from the alignments using the standard samtools-bcftools pipeline **[31].** This process led to 2,788,671 high quality SNPs with quality scores of at least 30 (1 error per 1000bp) and perfect trio-consistent genotype calls. Haplotypes were phased from genotypes using SHAPEIT (www.shapeit.fr; default settings). Structural variants, including duplications, deletions, insertions and inversions, were identified within the secondary ‘Trios’ dataset for candidate regions using DELLY version v0.7.3 **[32].** This software was applied using default settings, and its use in pipelines has been shown to reliably uncover structural variants from the 1000 Genomes Project, and validation experiments of randomly selected deletion loci show a high specificity **[32]**. Structural variants greater than 100,000 basepairs in length were removed to conservatively exclude false positives.

To increase genome-wide SNP resolution, our initial case-control dataset was imputed using a combined reference panel of the Phase 3 1000 Genomes project **[10]** and children within the trio dataset, using Beagle 4.1 **[11].** This allowed for the inclusion of 13.5 million additional high quality SNPs, to a total of 15.2 million SNPs. A total of 621,019 SNPs were removed from the pre-imputation dataset due to evidence of: (i) deviations in genotypic frequencies from Hardy-Weinberg equilibrium (HWE) as assessed using a chi-square test (>0.0001); (ii) high genotype call missingness (>10%); or (iii) low minor allele frequency (<0.01). 51 individuals were removed due to: (i) genotypic missingness (>0.1); (ii) abnormal PCA clustering or (iii) missing malaria phenotype data. 849,134 strand flips were identified with snpflip, with these being corrected pre-imputation with Plink v1.07. Raw hybridisation plots were manually verified for all top non-imputed GWAS associations, excluding *rs334* for which the data was unavailable. Linkage disequilibrium between SNPs in close genomic distance was calculated using Plink v1.07 **[33]**.

Targeted imputation was performed for HLA haplotypes within the major histocompatibility complex using 9,785 high quality SNPs within the region; for this we utilised SNP2HLA software (version 1.0.3) and the default Caucasian reference panel **[34]**. Association tests for this targeted analysis were performed through the pipeline described above. Similarly, 1,202 structural variants (698 deletions, 311 duplications, 19 insertions, 174 inversions) within chromosome four were imputed into our primary ‘case-control’ dataset using IMPUTE2 with default parameters, akin to standard SNP imputation. This approach allowed us to perform association analysis on those structural variants using EMMAX mixed model regression **[12]**. Trio parental SNP data was also used to provide additional context for our case-control SNPs within the wider Tanzanian population, as seen in **S1 Table.**

### Association analysis

Case-Control association analysis of SNPs was undertaken with EMMAX mixed model regression **[12],** controlling for age as a fixed effect and relatedness (represented by a kinship matrix) as a random effect (to reduce associations relating to familial clustering). Several genotypic models were implemented separately, including additive, heterozygous, dominant and recessive. Minimum P values from each model were utilised for top hit identification. Odds ratios were estimated with Plink v1.07 **[33]**. Our complementary conditional GWAS shared the pipeline for the main GWAS, but with HbS status added as an additional covariate. To evaluate the statistical potential of our GWAS study, we performed a retrospective power calculation (using http://zzz.bwh.harvard.edu/gpc/cc2.html). A study of 460 cases and 460 controls can detect odds ratios of at least 2 for a high risk allele minor allele frequency of 5% with a statistical power of 85% (and type I error of 10^−6^). A significance threshold of 10^−6^ was established using a permutation approach **[35]**. In particular, both the case-control status of the chromosomes were randomly permuted 10,000 times. From each of the 10,000 random experiments, we determined the maximum chi-square statistics (across the four genotypic tests) over all SNPs genotyped. We ordered these statistics and then calculated the 95 percentile. This was the estimate of the 0.05 significance level for the experiment performed, assuming inference is taken with respect to maximum chi-square statistic observed over all genotyped SNPs, and accounts for the linkage disequilibrium between SNPs and correlation between the results from applying the 4 genotypic tests.

### Selection analysis

Whole population Integrated Haplotype Scores (iHS) **[13]** and case-control Cross-Population Extended Haplotype Homozygosity (XP-EHH) **[14]** were calculated and normalised over the whole genome using selscan and norm **[36].** Core SNPs with a minor allele frequency below 0.01 were excluded from this analysis. In this context, high iHS values indicate a whole population selection signal whilst positive XP-EHH values indicate relative selection within the control population and negative XP-EHH values indicate relative selection within the case population. We looked for structural variants in regions with SNP-based signals of positive selection, as it possible that selection may actually be driven by structural variants (see **[37]** for an example).

## Supporting information

S1 Fig Population structure. Visualisation of the first two principal components, by (a) case-control status and (b) father’s ethnicity, highlights the existence of cryptic relatedness; (c) Principal component analysis reveals that the ‘Trios’ and primary ‘Case-Control’ participants overlap and are within the African cluster of the 1000 Genomes dataset; (d) Quantile-quantile plot for the observed and expected P values of the heterozygous model genome-wide association statistic.
(PNG)

S1 Table Full list of significant SNP associations, including odds ratios and minor allele frequencies. (DOCX)

S2 Table Structural variation identified within regions consisting of GWAS associations, known malaria candidates and sites under selection (iHS, XP-EHH). (DOCX)

S3 Table Candidate SNP associations. (DOCX)

S4 Table Regions under potential whole population positive selection (absolute iHS > 4). (DOCX)

S5 Table Regions under potential differential selection between cases and controls (absolute XP-EHH > 4). (DOCX)
