# Supplementary figures and images for: Novel genetic polymorphisms associated with severe malaria and under selective pressure in North-eastern Tanzania
Source: PLoS Genet. 2018 Jan 30;14(1):e1007172. doi: 10.1371/journal.pgen.1007172 (PMC5806895; doi:10.1371/journal.pgen.1007172)

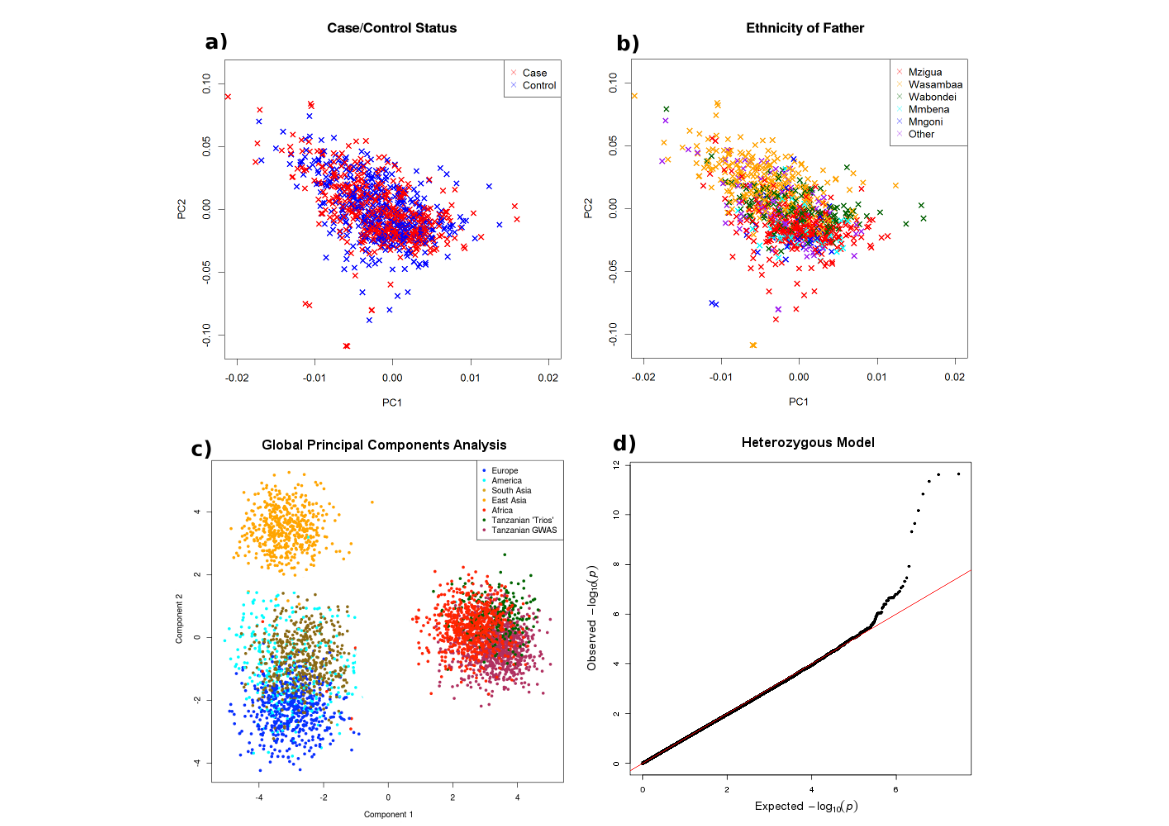

Supplement: S1 Fig — Visualisation of the first two principal components, by (a) case-control status and (b) father’s ethnicity, highlights the existence of cryptic relatedness; (c) Principal component analysis reveals that the ‘Trios’ and primary ‘Case-Control’ participants overlap and are within the African cluster of the 1000 Genomes dataset; (d) Quantile-quantile plot for the observed and expected P values of the heterozygous model genome-wide association statistic. (PNG) [file pgen.1007172.s001.png]
