# Supplementary material for: Novel genetic polymorphisms associated with severe malaria and under selective pressure in North-eastern Tanzania
Source: PLoS Genet. 2018 Jan 30;14(1):e1007172. doi: 10.1371/journal.pgen.1007172 (PMC5806895; doi:10.1371/journal.pgen.1007172)
Supplement: S1 Table — (DOCX) [file pgen.1007172.s002.docx]

**S1 Table: Full list of significant SNP associations (P < 1_x10_^-6^), including odds ratios and minor allele frequencies**

| **SNP** | **Gene** | **Location** | **Min P** | **Model** | **Conditional** | **Subtype** | **Odds Ratio** | **Case MAF** | **Control MAF** | **Trios MAF** | **African MAF** | **Global MAF** |
| --- | --- | --- | --- | --- | --- | --- | --- | --- | --- | --- | --- | --- |
| rs334 | HbS (in HBB) | 11:5248232 | 8.59E-13 | Heterozygous | - | HL: 1.81e-09 | 0.131 | 0.018 | 0.080 | 0.026 | 0.100 | 0.027 |
| rs113892119 | *HBB* | 11:5273865 | 1.43E-12 | Heterozygous | DOM: 2.63e-03 | HL: 3.25e-08 | 0.115 | 0.018 | 0.079 | 0.006 | 0.017 | 0.005 |
| rs141845179 | *HBB* | 11:5244665 | 2.67E-12 | Heterozygous | DOM: 8.99e-03 | HL: 2.20e-08 | 0.146 | 0.024 | 0.089 | 0.008 | 0.026 | 0.008 |
| rs201250014 | *HBB* | 11:5118578 | 4.55E-12 | Heterozygous | DOM: 5.25e-02 | HL: 3.53e-09 | 0.118 | 0.013 | 0.076 | 0.006 | 0.017 | 0.006 |
| rs142667079 | *HBB* | 11:5190723 | 8.46E-12 | Heterozygous | DOM: 5.25e-02 | HL: 5.39e-09 | 0.121 | 0.013 | 0.075 | 0.006 | 0.017 | 0.005 |
| rs113850170 | *HBB* | 11:5243936 | 4.68E-11 | Heterozygous | DOM: 1.30e-03 | HL: 6.00e-08 | 0.187 | 0.029 | 0.090 | 0.010 | 0.034 | 0.010 |
| rs145843585 | *HBB* | 11:5321510 | 3.53E-09 | Heterozygous | DOM: 8.70e-03 | HL: 1.78e-06 | 0.244 | 0.031 | 0.087 | 0.010 | 0.035 | 0.010 |
| rs12292063 | *HBB* | 11:5302406 | 6.21E-09 | Heterozygous | DOM: 1.61e-03 | CM: 5.89e-06 | 0.320 | 0.051 | 0.106 | 0.015 | 0.051 | 0.014 |
| rs111565479 | *HBB* | 11:5304648 | 3.47E-08 | Heterozygous | ADD: 1.52e-03 | CM: 4.75e-05 | 0.260 | 0.035 | 0.080 | 0.014 | 0.047 | 0.013 |
| rs138355232 | *HBB* | 11:5283010 | 7.53E-08 | Heterozygous | DOM: 8.99e-03 | HL: 2.74e-05 | 0.284 | 0.036 | 0.086 | 0.013 | 0.042 | 0.012 |
| rs112075505 | *HBB* | 11:5276402 | 8.70E-08 | Heterozygous | ADD: 7.63e-03 | HL: 2.27e-05 | 0.294 | 0.036 | 0.086 | 0.013 | 0.043 | 0.012 |
| rs112035597 | *HBB* | 11:5277116 | 8.70E-08 | Heterozygous | ADD: 7.63e-03 | HL: 2.27e-05 | 0.294 | 0.036 | 0.086 | 0.013 | 0.043 | 0.012 |
| rs113981422 | *HBB* | 11:5277117 | 8.70E-08 | Heterozygous | ADD: 7.63e-03 | HL: 2.27e-05 | 0.294 | 0.036 | 0.086 | 0.013 | 0.043 | 0.012 |
| rs28674962 | *HBB* | 11:5351215 | 1.83E-07 | Heterozygous | DOM: 3.66e-02 | RD: 6.50e-05 | 0.223 | 0.019 | 0.065 | 0.012 | 0.039 | 0.011 |
| rs145430058 | *HBB* | 11:5037158 | 9.23E-07 | Heterozygous | DOM: 2.47e-01 | HL: 1.20e-06 | 0.197 | 0.011 | 0.048 | 0.003 | 0.008 | 0.003 |
| rs148767281 | *HBB* | 11:5053696 | 9.23E-07 | Heterozygous | DOM: 2.47e-01 | HL: 1.20e-06 | 0.197 | 0.011 | 0.048 | 0.003 | 0.007 | 0.002 |
| rs149827427 | *HBB* | 11:5059256 | 9.23E-07 | Heterozygous | DOM: 2.47e-01 | HL: 1.20e-06 | 0.197 | 0.011 | 0.048 | 0.003 | 0.007 | 0.002 |
| rs139248000 | *HBB* | 11:5067053 | 9.23E-07 | Heterozygous | DOM: 2.47e-01 | HL: 1.20e-06 | 0.197 | 0.011 | 0.048 | 0.003 | 0.007 | 0.002 |
| rs191914703 | *HBB* | 11:5087315 | 9.23E-07 | Heterozygous | DOM: 2.47e-01 | HL: 1.20e-06 | 0.197 | 0.011 | 0.048 | 0.003 | 0.007 | 0.003 |
| rs147924587 | *HBB* | 11:5089198 | 9.23E-07 | Heterozygous | DOM: 2.47e-01 | HL: 1.20e-06 | 0.197 | 0.011 | 0.048 | 0.003 | 0.007 | 0.002 |
| rs12295158 | *HBB* | 11:5252794 | 9.47E-07 | Heterozygous | DOM: 1.49e-01 | SMA: 2.23e-04 | 0.328 | 0.052 | 0.110 | 0.015 | 0.048 | 0.014 |
| rs184434980 | *HBB* | 11:4995964 | 1.24E-06 | Heterozygous | DOM: 2.47e-01 | HL: 5.49e-07 | 0.229 | 0.015 | 0.051 | 0.003 | 0.006 | 0.002 |
| rs74765645 | *HBB* | 11:5211496 | 1.89E-06 | Heterozygous | DOM: 1.21e-02 | SMA: 6.53e-06 | 0.369 | 0.056 | 0.104 | 0.019 | 0.059 | 0.017 |
| rs143802965 | *HBB* | 11:4986124 | 1.98E-06 | Heterozygous | DOM: 2.47e-01 | HL: 4.24e-07 | 0.251 | 0.016 | 0.052 | 0.003 | 0.006 | 0.002 |
| rs143210906 | *HBB* | 11:4994082 | 2.11E-06 | Heterozygous | DOM: 2.47e-01 | HL: 8.21e-07 | 0.233 | 0.015 | 0.050 | 0.002 | 0.005 | 0.002 |
| rs115909008 | *HBB* | 11:4994513 | 2.11E-06 | Heterozygous | DOM: 2.47e-01 | HL: 8.21e-07 | 0.233 | 0.015 | 0.050 | 0.002 | 0.005 | 0.002 |
| rs140790233 | *HBB* | 11:4998510 | 2.11E-06 | Heterozygous | DOM: 2.47e-01 | HL: 8.21e-07 | 0.233 | 0.015 | 0.050 | 0.002 | 0.005 | 0.002 |
| rs11036485 | *HBB* | 11:5279040 | 2.43E-06 | Heterozygous | HET: 5.88e-02 | HL: 1.21e-05 | 0.480 | 0.138 | 0.202 | 0.037 | 0.122 | 0.033 |
| rs11036493 | *HBB* | 11:5279816 | 2.43E-06 | Heterozygous | HET: 5.88e-02 | HL: 1.21e-05 | 0.480 | 0.138 | 0.202 | 0.037 | 0.123 | 0.033 |
| rs10128653 | *HBB* | 11:5277461 | 2.91E-06 | Heterozygous | HET: 6.10e-02 | HL: 1.19e-05 | 0.481 | 0.143 | 0.205 | 0.037 | 0.123 | 0.033 |
| rs145099017 | *HBB* | 11:5338232 | 3.61E-06 | Heterozygous | DOM: 1.39e-02 | HL: 4.32e-04 | 0.288 | 0.022 | 0.064 | 0.012 | 0.042 | 0.011 |
| rs141413067 | *HBB* | 11:5010121 | 3.75E-06 | Heterozygous | DOM: 2.47e-01 | HL: 2.87e-06 | 0.233 | 0.013 | 0.048 | 0.002 | 0.005 | 0.002 |
| rs183294476 | *HBB* | 11:5012063 | 3.75E-06 | Heterozygous | DOM: 2.47e-01 | HL: 2.87e-06 | 0.233 | 0.013 | 0.048 | 0.003 | 0.005 | 0.003 |
| rs1973075 | *HBB* | 11:5012257 | 3.75E-06 | Heterozygous | DOM: 2.47e-01 | HL: 2.87e-06 | 0.233 | 0.013 | 0.048 | 0.002 | 0.005 | 0.002 |
| rs142527103 | *HBB* | 11:5012800 | 3.75E-06 | Heterozygous | DOM: 2.47e-01 | HL: 2.87e-06 | 0.233 | 0.013 | 0.048 | 0.003 | 0.007 | 0.002 |
| rs111978456 | *HBB* | 11:5311492 | 4.43E-06 | Heterozygous | DOM: 6.19e-03 | RD: 8.49e-04 | 0.411 | 0.054 | 0.100 | 0.016 | 0.056 | 0.016 |
| rs16912210 | *HBB* | 11:5263853 | 5.60E-06 | Heterozygous | DOM: 4.86e-04 | HL: 2.71e-05 | 0.445 | 0.124 | 0.174 | 0.055 | 0.091 | 0.052 |
| rs150640479 | *HBB* | 11:5283777 | 7.56E-06 | Heterozygous | DOM: 8.01e-02 | HL: 2.07e-05 | 0.493 | 0.144 | 0.205 | 0.037 | 0.123 | 0.033 |
| rs77333754 | *HBB* | 11:5023083 | 7.80E-06 | Heterozygous | DOM: 2.47e-01 | HL: 3.32e-06 | 0.226 | 0.012 | 0.046 | 0.007 | 0.006 | 0.006 |
| rs146877463 | *HBB* | 11:4984021 | 9.55E-06 | Heterozygous | DOM: 2.47e-01 | HL: 3.05e-06 | 0.310 | 0.019 | 0.053 | 0.003 | 0.006 | 0.002 |
| rs9296359 | *TREML4* | 6:41205690 | 1.21E-07 | Heterozygous | HET: 4.42e-07 | SMA: 3.29e-07 | 4.087 | 0.084 | 0.024 | 0.245 | 0.037 | 0.253 |
| rs149085856 | Intergenic (LINC00670) | 17:12399526 | 2.15E-07 | Additive | ADD: 1.06e-06 | HL: 2.81e-07 | 3.867 | 0.067 | 0.016 | 0.021 | 0.069 | 0.019 |
| rs113449872 | Intergenic | 5:43909343 | 2.17E-07 | Heterozygous | HET: 2.93e-07 | SMA: 2.92e-05 | 0.354 | 0.075 | 0.144 | 0.043 | 0.123 | 0.043 |
| rs182627261 | Intergenic | 5:43912529 | 2.17E-07 | Heterozygous | HET: 2.93e-07 | SMA: 2.92e-05 | 0.354 | 0.075 | 0.144 | 0.043 | 0.123 | 0.043 |
| rs143410369 | Intergenic | 5:43912572 | 2.17E-07 | Heterozygous | HET: 2.93e-07 | SMA: 2.92e-05 | 0.354 | 0.075 | 0.144 | 0.043 | 0.123 | 0.043 |
| rs72762400 | Intergenic | 5:43921648 | 2.26E-07 | Heterozygous | HET: 2.36e-07 | SMA: 8.43e-06 | 0.361 | 0.081 | 0.150 | 0.044 | 0.126 | 0.044 |
| rs4568372 | Intergenic | 5:43930034 | 2.26E-07 | Heterozygous | HET: 2.36e-07 | SMA: 8.43e-06 | 0.361 | 0.081 | 0.150 | 0.044 | 0.126 | 0.044 |
| rs72762397 | Intergenic | 5:43908543 | 2.83E-07 | Heterozygous | HET: 3.93e-07 | SMA: 3.31e-05 | 0.358 | 0.075 | 0.143 | 0.042 | 0.123 | 0.042 |
| rs72762398 | Intergenic | 5:43908680 | 2.83E-07 | Heterozygous | HET: 3.93e-07 | SMA: 3.31e-05 | 0.358 | 0.075 | 0.143 | 0.042 | 0.123 | 0.042 |
| rs72762396 | Intergenic | 5:43906364 | 2.92E-07 | Heterozygous | HET: 3.16e-07 | SMA: 9.60e-06 | 0.365 | 0.081 | 0.149 | 0.043 | 0.126 | 0.043 |
| rs7444352 | Intergenic | 5:43946096 | 3.95E-07 | Heterozygous | HET: 4.82e-07 | SMA: 1.62e-05 | 0.349 | 0.079 | 0.147 | 0.043 | 0.125 | 0.043 |
| rs141375659 | Intergenic | 5:43937743 | 4.10E-07 | Heterozygous | HET: 4.69e-07 | SMA: 1.23e-05 | 0.365 | 0.081 | 0.148 | 0.043 | 0.126 | 0.043 |
| rs72762392 | Intergenic | 5:43898908 | 5.80E-07 | Heterozygous | HET: 7.20e-07 | SMA: 1.20e-05 | 0.367 | 0.080 | 0.146 | 0.041 | 0.119 | 0.041 |
| rs116185108 | Intergenic | 5:43963434 | 6.54E-07 | Heterozygous | HET: 2.10e-06 | SMA: 2.25e-05 | 0.367 | 0.078 | 0.139 | 0.043 | 0.108 | 0.043 |
| rs144955924 | Intergenic | 5:43964366 | 6.54E-07 | Heterozygous | HET: 2.10e-06 | SMA: 2.25e-05 | 0.367 | 0.078 | 0.139 | 0.043 | 0.109 | 0.043 |
| rs72762389 | Intergenic | 5:43892232 | 7.65E-07 | Recessive | REC: 1.94e-06 | SMA: 1.44e-05 | 2.624 | 0.070 | 0.140 | 0.038 | 0.107 | 0.038 |
| rs72762395 | Intergenic | 5:43904012 | 1.22E-06 | Recessive | HET: 2.21e-06 | SMA: 2.93e-05 | 2.611 | 0.072 | 0.140 | 0.040 | 0.115 | 0.040 |
| rs72764224 | Intergenic | 5:43948071 | 7.60E-06 | Heterozygous | HET: 2.22e-05 | SMA: 8.42e-05 | 0.374 | 0.073 | 0.131 | 0.041 | 0.100 | 0.041 |
| rs112283878 | Intergenic | 5:43950047 | 7.60E-06 | Heterozygous | HET: 2.22e-05 | SMA: 8.42e-05 | 0.374 | 0.073 | 0.131 | 0.041 | 0.100 | 0.041 |
| rs72764227 | Intergenic | 5:43951609 | 7.60E-06 | Heterozygous | HET: 2.22e-05 | SMA: 8.42e-05 | 0.374 | 0.073 | 0.131 | 0.041 | 0.099 | 0.041 |
| rs146640417 | Intergenic | 5:43957834 | 7.87E-06 | Heterozygous | HET: 2.16e-05 | SMA: 6.47e-05 | 0.392 | 0.075 | 0.132 | 0.041 | 0.101 | 0.041 |
| rs201594601 | Intergenic | 5:43958015 | 7.87E-06 | Heterozygous | HET: 2.16e-05 | SMA: 6.47e-05 | 0.392 | 0.075 | 0.132 | 0.041 | 0.101 | 0.041 |
| rs11335470 | *LINC00944* | 12:127237620 | 2.52E-07 | Heterozygous | HET: 1.86e-06 | HL: 9.04e-05 | 0.403 | 0.053 | 0.118 | 0.062 | 0.911 | 0.061 |
| rs1205303 | *LINC00944* | 12:127232689 | 4.10E-07 | Recessive | REC: 3.00e-06 | HL: 1.28e-04 | 2.391 | 0.054 | 0.119 | 0.061 | 0.912 | 0.939 |
| rs1798817 | *LINC00944* | 12:127239061 | 5.57E-07 | Heterozygous | HET: 2.79e-06 | HL: 3.52e-04 | 0.408 | 0.054 | 0.116 | 0.053 | 0.915 | 0.948 |
| rs73832816 | *C4orf17* | 4:100429757 | 3.75E-07 | Recessive | REC: 9.48e-07 | CM: 1.02e-06 | 0.289 | 0.085 | 0.025 | 0.010 | 0.035 | 0.009 |
| rs17624383 | Intergenic | 7:53676837 | 5.62E-07 | Additive | ADD: 3.28e-06 | RD: 4.61e-07 | 2.500 | 0.130 | 0.066 | 0.109 | 0.045 | 0.111 |
| rs28852695 | Intergenic | 7:53673564 | 3.35E-06 | Additive | ADD: 1.69e-05 | RD: 2.16e-06 | 2.322 | 0.136 | 0.073 | 0.110 | 0.045 | 0.111 |
| rs2194642 | Intergenic | 7:53666370 | 6.66E-06 | Additive | ADD: 3.16e-05 | RD: 6.80e-06 | 2.272 | 0.135 | 0.075 | 0.109 | 0.046 | 0.111 |
| rs2967790 | *KLHL3, MYOT* | 5:137011761 | 5.85E-07 | Additive | ADD: 2.46e-06 | HL: 8.65e-06 | 0.590 | 0.252 | 0.367 | 0.147 | 0.272 | 0.141 |
| rs2967793 | *KLHL3, MYOT* | 5:137015272 | 9.83E-07 | Additive | ADD: 3.24e-06 | HL: 1.81e-06 | 0.591 | 0.246 | 0.358 | 0.146 | 0.269 | 0.140 |
| rs2905584 | *KLHL3, MYOT* | 5:137004506 | 1.22E-06 | Additive | ADD: 4.01e-06 | HL: 1.85e-05 | 0.594 | 0.254 | 0.366 | 0.148 | 0.273 | 0.142 |
| rs138625444 | *KLHL3, MYOT* | 5:137005325 | 1.22E-06 | Additive | ADD: 4.01e-06 | HL: 1.85e-05 | 0.594 | 0.254 | 0.366 | 0.148 | 0.273 | 0.142 |
| rs916861 | *KLHL3, MYOT* | 5:137005916 | 1.58E-06 | Additive | ADD: 5.14e-06 | HL: 1.85e-05 | 0.594 | 0.255 | 0.366 | 0.148 | 0.273 | 0.142 |
| rs2349033 | *KLHL3, MYOT* | 5:137006016 | 1.58E-06 | Additive | ADD: 5.14e-06 | HL: 1.85e-05 | 0.594 | 0.255 | 0.366 | 0.148 | 0.273 | 0.142 |
| rs11386045 | *KLHL3, MYOT* | 5:137006018 | 1.58E-06 | Additive | ADD: 5.14e-06 | HL: 1.85e-05 | 0.594 | 0.255 | 0.366 | 0.148 | 0.273 | 0.142 |
| rs2967789 | *KLHL3, MYOT* | 5:137006841 | 1.58E-06 | Additive | ADD: 5.14e-06 | HL: 1.85e-05 | 0.594 | 0.255 | 0.366 | 0.148 | 0.273 | 0.142 |
| rs2905585 | *KLHL3, MYOT* | 5:137007200 | 1.58E-06 | Additive | ADD: 5.14e-06 | HL: 1.85e-05 | 0.594 | 0.255 | 0.366 | 0.148 | 0.273 | 0.142 |
| rs2905586 | *KLHL3, MYOT* | 5:137007745 | 1.58E-06 | Additive | ADD: 5.14e-06 | HL: 1.85e-05 | 0.594 | 0.255 | 0.366 | 0.148 | 0.273 | 0.142 |
| rs3756687 | *KLHL3, MYOT* | 5:137201693 | 5.03E-06 | Additive | ADD: 1.37e-05 | HL: 9.43e-06 | 0.578 | 0.191 | 0.291 | 0.127 | 0.201 | 0.123 |
| rs60701788 | *KLHL3, MYOT* | 5:137132514 | 5.50E-06 | Additive | ADD: 1.51e-05 | HL: 1.69e-05 | 0.586 | 0.192 | 0.291 | 0.120 | 0.201 | 0.116 |
| rs7722600 | *KLHL3, MYOT* | 5:137194762 | 5.81E-06 | Additive | ADD: 1.90e-05 | HL: 1.44e-05 | 0.585 | 0.192 | 0.292 | 0.130 | 0.213 | 0.126 |
| rs144312179 | *FAM155A* | 13:108228013 | 6.24E-07 | Additive | ADD: 2.92e-06 | HL: 1.35e-06 | 0.207 | 0.011 | 0.052 | 0.007 | 0.021 | 0.006 |
| rs112630202 | *FAM155A* | 13:108215975 | 3.51E-06 | Heterozygous | REC: 1.41e-05 | HL: 3.46e-04 | 0.098 | 0.003 | 0.032 | 0.008 | 0.023 | 0.007 |
| rs112240018 | *FAM155A* | 13:108215989 | 3.51E-06 | Heterozygous | REC: 1.41e-05 | HL: 3.46e-04 | 0.098 | 0.003 | 0.032 | 0.008 | 0.023 | 0.007 |
| rs374297923 | *FAM155A* | 13:108220281 | 3.51E-06 | Heterozygous | REC: 1.41e-05 | HL: 3.46e-04 | 0.098 | 0.003 | 0.032 | 0.007 | 0.020 | 0.006 |
| rs185820024 | *FAM155A* | 13:108222323 | 4.59E-06 | Additive | REC: 1.99e-05 | HL: 3.53e-04 | 0.101 | 0.003 | 0.031 | 0.010 | 0.028 | 0.010 |
| rs183246449 | *FAM155A* | 13:108213299 | 6.90E-06 | Additive | HET: 2.97e-05 | HL: 5.48e-04 | 0.102 | 0.003 | 0.031 | 0.007 | 0.020 | 0.006 |
| rs114169033 | AF146191.4-004 (lincRNA) | 4:190717704 | 6.67E-07 | Additive | ADD: 1.30e-06 | RD: 5.62e-07 | 3.326 | 0.103 | 0.041 | 0.019 | 0.064 | 0.017 |
| rs138239288 | AF146191.4-004 (lincRNA) | 4:190715758 | 1.85E-06 | Additive | ADD: 3.59e-06 | RD: 1.82e-06 | 3.236 | 0.101 | 0.042 | 0.019 | 0.065 | 0.018 |
| rs114925393 | AF146191.4-004 (lincRNA) | 4:190717243 | 1.85E-06 | Additive | ADD: 3.59e-06 | RD: 1.82e-06 | 3.236 | 0.101 | 0.042 | 0.019 | 0.066 | 0.018 |
| rs6682413 | *IL23R, IL12RB2* | 1:67731614 | 7.98E-07 | Recessive | REC: 1.03e-06 | SMA: 1.23e-04 | 0.479 | 0.361 | 0.273 | 0.289 | 0.371 | 0.288 |
| rs6588252 | *IL23R, IL12RB2* | 1:67739028 | 1.07E-06 | Recessive | REC: 9.00e-07 | SMA: 5.86e-05 | 0.486 | 0.356 | 0.271 | 0.290 | 0.371 | 0.289 |
| rs11209031 | *IL23R, IL12RB2* | 1:67739588 | 1.07E-06 | Recessive | REC: 9.00e-07 | SMA: 5.86e-05 | 0.486 | 0.355 | 0.271 | 0.290 | 0.371 | 0.289 |
| rs6677188 | *IL23R, IL12RB2* | 1:67740403 | 1.62E-06 | Recessive | REC: 1.48e-06 | SMA: 9.83e-05 | 0.495 | 0.357 | 0.273 | 0.292 | 0.377 | 0.291 |
| rs59384635 | *IL23R, IL12RB2* | 1:67738621 | 2.78E-06 | Heterozygous | HET: 1.04e-05 | SMA: 4.01e-04 | 0.535 | 0.217 | 0.163 | 0.241 | 0.199 | 0.241 |
| rs12045232 | *IL23R, IL12RB2* | 1:67738359 | 4.26E-06 | Heterozygous | HET: 1.54e-05 | SMA: 4.01e-04 | 0.541 | 0.216 | 0.163 | 0.239 | 0.198 | 0.240 |
| rs10889679 | *IL23R, IL12RB2* | 1:67752088 | 7.53E-06 | Recessive | REC: 3.08e-06 | SMA: 8.78e-05 | 0.502 | 0.378 | 0.306 | 0.317 | 0.388 | 0.316 |
| rs73505850 | *CSMD1* | 8:4754838 | 7.98E-07 | Additive | ADD: 1.42e-06 | SMA: 1.20e-05 | 4.795 | 0.052 | 0.013 | 0.017 | 0.015 | 0.017 |
| rs73503859 | *CSMD1* | 8:4724338 | 1.78E-06 | Additive | ADD: 5.06e-06 | HL: 2.14e-05 | 3.579 | 0.069 | 0.025 | 0.015 | 0.026 | 0.015 |
| rs10435673 | *CSMD1* | 8:4736407 | 3.72E-06 | Additive | ADD: 1.20e-05 | SMA: 9.58e-05 | 3.752 | 0.056 | 0.019 | 0.015 | 0.974 | 0.985 |
| rs73503887 | *CSMD1* | 8:4733991 | 3.72E-06 | Additive | ADD: 1.20e-05 | SMA: 9.58e-05 | 3.752 | 0.056 | 0.019 | 0.013 | 0.018 | 0.013 |
| rs4875122 | *CSMD1* | 8:4740387 | 8.18E-06 | Additive | ADD: 1.45e-05 | SMA: 3.78e-05 | 4.026 | 0.049 | 0.014 | 0.018 | 0.980 | 0.982 |
| rs8109875 | *ZNF536* | 19:31069639 | 8.69E-07 | Recessive | REC: 3.57e-06 | SMA: 2.80e-05 | 0.507 | 0.432 | 0.323 | 0.146 | 0.432 | 0.136 |
| rs1878468 | AC108142.1 (antisense) | 4:182822332 | 8.98E-07 | Heterozygous | HET: 1.19e-06 | HL: 8.10e-07 | 0.383 | 0.062 | 0.111 | 0.109 | 0.057 | 0.111 |
| rs3133394 | Intergenic | 11:130417522 | 9.41E-07 | Additive | ADD: 1.08e-06 | CM: 9.49e-06 | 0.551 | 0.196 | 0.302 | 0.124 | 0.283 | 0.119 |
| rs373636663 | Intergenic | 11:130417624 | 1.85E-06 | Additive | ADD: 1.80e-06 | CM: 1.40e-05 | 0.568 | 0.206 | 0.313 | 0.131 | 0.309 | 0.127 |
| rs992330 | Intergenic | 11:130422569 | 6.53E-06 | Additive | ADD: 1.45e-05 | HL: 3.11e-05 | 0.613 | 0.238 | 0.342 | 0.137 | 0.333 | 0.133 |
| rs2703755 | Intergenic | 11:130424729 | 8.56E-06 | Additive | ADD: 1.86e-05 | HL: 3.11e-05 | 0.618 | 0.239 | 0.342 | 0.138 | 0.335 | 0.133 |

Allele models: ADD Additive, HET Heterozygous, DOM Dominant, REC Recessive. Subtype significances: HL Hyperlactatemia; SMA Severe Malarial Anaemia; RD Respiratory Distress; CM Cerebral Malaria. Locations correspond to the GRCh37 reference genome. Case, Control, and Trio minor allele frequencies (MAFs) are from the Tanzanian GWAS and Trio datasets (excluding children); MAFs for 1000 Genomes populations are presented for African and Global populations. Minimum P indicates the most significant P for feature within the case-control GWAS, whilst Condition and Subtype Ps indicate the most significant P value for those SNPs when controlling for rs334 status, or considering the severe malarial subtypes.
