# Supplementary material for: Novel genetic polymorphisms associated with severe malaria and under selective pressure in North-eastern Tanzania
Source: PLoS Genet. 2018 Jan 30;14(1):e1007172. doi: 10.1371/journal.pgen.1007172 (PMC5806895; doi:10.1371/journal.pgen.1007172)
Supplement: S2 Table — (DOCX) [file pgen.1007172.s003.docx]

**S2 Table: Structural variation identified within** **regions consisting of GWAS associations, known malaria candidates and sites under selection (iHS, XP-EHH)**

| **Gene/s** | **Region of Interest*** | **Reason** | **Number of unique forms in Trios (total count)** | | | | | **Presence in 1000 Genomes populations** |
| --- | --- | --- | --- | --- | --- | --- | --- | --- |
|  |  |  | **Total** | **Del.** | **Dup.** | **Ins.** | **Inv.** |  |
| *IL23R and IL12RB2* | 1: 67,632,083-67,862,583 | GWAS | 1  (1) | 1  (1) | - | - | - | 1 deletion; 1 total case  (1 East Asian) |
| *RYR2* | 1: 237,205,505-237,997,288 | GWAS | 49  (51) | 9  (11) | - | - | 40  (41) | 8 deletions; 23 total cases  (14 Africans, 6 East Asians, 2 Americans, 1 European |
| *MAP1B* | 5: 71,403,061-71,505,395 | GWAS | 2  (9) | 2  (9) | - | - | - | - |
| *KLHL3* | 5: 136,953,189-137,071,779 | GWAS | 63  (153) | 63  (153) | - | - | - | 1 deletion; 15 total cases  (3 South Asians, 9 Europeans, 3 Americans) |
| *HBB* | 11: 5,246,694-5,250,625 | GWAS/ Candidate | - | - | - | - | - | 4 deletions; 14 total cases  (3 East Asians, 6 African) |
| *LINC00943/4* | 12: 127,214,333-127,256,957 | GWAS | - | - | - | - | - | 2 deletions; 3 total cases  (1 South Asian, 1 African, 1 East Asian) |
| *LIPG* | 18: 47,087,069-47,119,272 | GWAS | 13  (13) | 13  (13) | - | - | - | - |
| *ZNF536* | 19: 30,719,197-31,204,445 | GWAS | 2  (3) | 2  (3) | - | - | - | 4 deletions, 1 ALU deletions; 200 total cases  (117 Europeans, 44 Americans, 32 South Asians, 6 Africans, 1 East Asian) |
| *ATP2B4* | 1: 203,595,689-203,713,209 | Candidate | 25  (25) | 25  (25) | - | - | - | 1 deletion; 857 total cases  (246 South Asian, 194 East Asian, 177 African, 159 European, 81 American) |
| *USP38* | 4: 144,106,070-144,144,983 | Candidate | - | - | - | - | - | - |
| *FREM3* | 4: 144,498,455-144,621,828 | Candidate | 28  (29) | 28  (29) | - | - | - | 1 deletion; 9 total cases  (4 East Asians, 5 Europeans) |
| *GYPE, GYPB, GYPA* | 4:144,792,020-145,061,904 | Candidate | 55  (59) | 31  (35) | 24  (24) | - | - | 8 deletions, 2 duplications; 133 total cases  (102 Africans, 9 South Asians, 7 Americans, 14 East Asians, 1 European) |
| *Major Histocompatibility Complex*  *(including HLA)* | 6: 28,477,796-33,448,353 | Candidate/iHS/XP-EHH | 2333  (3337) | 1711  (2552) | 1711  (2552) | 1711  (2552) | 1711  (2552) | 101 deletions, 13 duplications, 8 CNVs, 6 ALU deletions; 60,948 total cases  (all samples) |
| *ABO* | 9: 136,125,788-136,150,617 | Candidate | 9  (21) | 1  (1) | 6  (6) | 1  (13) | 1  (1) | 1 deletion; 20 total cases  (17 Africans, 3 American) |
| *MARVELD3* | 16: 71,660,064-71,676,017 | Candidate | 5  (5) | 5  (5) | - | - | - | 1 deletion; 4 total cases  (3 Africans, 1 American) |
| *HBA2* | 16: 222,846-223,709 | Candidate | 3  (3) | 3  (3) | - | - | - | 2 deletions; 274 total cases  (192 Africans, 35 East Asians, 23 South Asians, 17 Americans, 4 Europeans) |
| *HBA1* | 16: 226,679-227,521 | Candidate | 1  (1) | 1  (1) | - | - | - | 3 deletions; 297 total cases  (192 Africans, 25 East Asians, 23 South Asians, 17 American, 4 Europeans) |
| *RORC, C2CD4D, THEM5* | 1: 151,792,842-151,817,543 | iHS | 2  (2) | 2  (2) | - | - | - | - |
| *DUSP19, NUP35* | 2: 183,699,180-185,281,789 | iHS | 139  (280) | 101  (213) | - | - | 38  (67) | 27 deletions, 5 duplications, 1 CNV, 1 inversion; 1,302 total cases  (346 Africans, 209 Europeans, 202 East Asians, 185 Americans, 139 South Asians) |
| *ERBB4* | 2: 212,380,286-213,576,272 | iHS | 65  (236) | 59  (207) | - | 5  (28) | 1  (1) | 22 deletions, 7 duplications, 2 CNVs, 1 ALU deletion, 1 inversion, 1 insertions; 4,165 total cases  (all samples) |
| *MCUR1* | 6: 13,786,789-13,814,800 | XP-EHH | 2  (2) | 2  (2) | - | - | - | 1 deletions; 1 total case  (1 African) |
| *PPARD, MKRNP2, FANCE, TEAD3, RPL10A, TULP1, FKBP5, ARMC12* | 6: 35,337,931-35,732,137 | iHS | 101  (179) | 94  (170) | 3  (3) | 3  (5) | 1  (1) | 6 duplications, 4 deletions, 1 CNV; 2,806 total cases  (629 Africans, 473 East Asians, 451 Europeans, 443 South Asians, 332 Americans) |
| *GCLC* | 6: 53,362,139-53,481,768 | XP-EHH | 3  (4) | 3  (4) | - | - | - | 2 deletions, 3 total cases  (1 African, 2 Europeans) |
| *RIMS1* | 6: 72,805,811-72,828,559 | iHS | 1  (1) | 1  (1) | - | - | - | 1 duplication, 1 CNV; 35 total cases  (16 Europeans, 10 Americans, 6 South Asians, 2 Africans, 1 East Asian) |
| *POM121L12* | 7: 53,103,349-53,104,617 | XP-EHH | - | - | - | - | - | - |
| *SYNJ2BP, ADAM21, ADAM20* | 14: 70,838,148-71,001,732 | XP-EHH | 21  (21) | 21  (21) | - | - | - | 1 deletion; 1 total case  (1 East Asian) |
| *ZFHX3* | 16: 72,916,326-73,133,159 | iHS | 3  (3) | 3  (3) | - | - | - | 3 deletions; 5 total cases  (2 Europeans, 1 South Asian, 1 African, 1 East Asian) |
| *ITGAE* | 17: 3,632,836-3,689,132 | iHS | 3  (9) | 3  (9) | - | - | - | - |
| *ERG, ETS2* | 21: 39,751,949-40,196,879 | XP-EHH | 23  (41) | 21  (38) | - | 2  (3) | - | 7 deletions, 1 ALU deletion, 1 duplication; 2,727 total cases  (457 Europeans, 412 Africans, 379 South Asians, 296 Americans, 220 East Asians) |

* Locations correspond to the GRCh37 reference genome; Del. deletion; Dup. duplication; Ins. insertion; Inv. inversion
