# Supplementary material for: Novel genetic polymorphisms associated with severe malaria and under selective pressure in North-eastern Tanzania
Source: PLoS Genet. 2018 Jan 30;14(1):e1007172. doi: 10.1371/journal.pgen.1007172 (PMC5806895; doi:10.1371/journal.pgen.1007172)
Supplement: S3 Table — (DOCX) [file pgen.1007172.s004.docx]

**S3 Table: Candidate SNP Associations**

| **SNP ID** | **Gene** | **Location** | **Min. P** | **Model** | **Case MAFs** | **Control MAFs** | **Trios MAFs** | **Kenya**  **MAFs** | **Nigeria**  **MAFs** | **Global MAFs** |
| --- | --- | --- | --- | --- | --- | --- | --- | --- | --- | --- |
| *rs334* | *HBB* | 11:5248232 | 2.61_x10_^-13 *^ | Heterozygous | 0.020 | 0.080 | 0.026 | 0.101 | 0.139 | 0.027 |
| *rs4951074 rs10900585 rs55868763 rs1541255* | *ATP2B4* | 1:203660781  1:203654024  1:203652140  1:203652141 | 5.19_x10_^-1^  2.07_x10_^-1^  4.20_x10_^-1^  4.20_x10_^-1^ | Dominant  Additive  Dominant  Dominant | 0.323  0.338  0.330  0.330 | 0.340  0.375  0.354  0.354 | 0.285  0.327  0.298  0.297 | 0.374  0.414  0.394  0.394 | 0.421  0.481  0.417  0.417 | 0.152  0.172  0.150  0.150 |
| *rs149914432*  *rs186790584*  *rs186873296* | *FREM3* | 4:144666678  4:144680140  4:144702474 | 1.73_x10_^-2^  1.74_x10_^-2^  2.28_x10_^-2^ | Recessive | 0.032  0.031  0.030 | 0.058  0.054  0.053 | 0.024  0.024  0.024 | 0.015  0.005  0.005 | 0.005  0.005  0.005 | 0.002  0.001  0.001 |
| *rs4266246*  *rs28459062* | *USP38* | 4:143971242  4:144039139 | 2.21_x10_^-4^;  4.66_x10_^-2^ | Additive  Recessive | 0.316  0.150 | 0.229  0.184 | 0.285  0.126 | 0.258  0.207 | 0.204  0.213 | 0.438  0.092 |
| *Deletion* | *GYPE, B, A* | 4:144801719-145041744 | 3.01_x10_^-1^ | Additive | 0.001 | 0.000 | 0.004 | - | - | - |
| *rs1264362*  *rs2523589*  *HLA_B_07* | *HLA* | 6:30776590  6:31327334  6:31431272 | 1.90_x10_^-3^  9.33_x10_^-5^  6.49_x10_^-5^ | Additive  Dominant  Dominant | 0.200  0.394  0.394 | 0.246  0.483  0.484 | -  -  - | 0.131  0.288  - | 0.032  0.301  - | 0.153  0.356  - |
| *rs8176746*  *rs7853989*  *rs1053878*  *rs8176719* | *ABO* | 9:136131322  9:136131592  9:136131651  9:136132908 | 1.89_x10_^-1^  2.27_x10_^-1^  3.98_x10_^-1^  8.69_x10_^-2^ | Dominant  Dominant  Heterozygous  Additive | 0.164  0.166  0.261  0.326 | 0.149  0.155  0.267  0.294 | 0.156  0.158  0.292  0.311 | 0.162  0.172  0.202  0.288 | 0.171  0.171  0.231  0.231 | 0.153  0.164  0.133  0.344 |
| *rs2334880* | *MARVELD3* | 16:71653637 | 7.09_x10_^-1^ | Dominant | 0.446 | 0.449 | 0.441 | 0.470 | 0.407 | 0.157 |
| *α-thalassemia deletion* | *HBA1, HBA2* | - | 1.80_x10_^-2^ | Recessive | 0.264 | 0.318 | - | - | - | - |

* Hyperlactatemia, severe malarial anaemia; - alleles not identified in a dataset; minor allele frequencies (MAFs) for 1000 Genomes populations are presented (Kenya - Luhya; Nigeria – Yoruba; Global – overall); other candidates include: rs12083094 (*PAPPA2*, P = 3.13_x10_^-3^), rs73498006 (*DPP6*, P = 2.41 _x10_^-2^), rs2985337 (*EPB41*, P = 3.56 _x10_^-2^), rs149373719 (*INPP4B*, P = 5.58 _x10_^-2^)
