# Supplementary material for: Novel genetic polymorphisms associated with severe malaria and under selective pressure in North-eastern Tanzania
Source: PLoS Genet. 2018 Jan 30;14(1):e1007172. doi: 10.1371/journal.pgen.1007172 (PMC5806895; doi:10.1371/journal.pgen.1007172)
Supplement: S4 Table — (DOCX) [file pgen.1007172.s005.docx]

**S4 Table: Regions under potential whole population positive selection (absolute iHS > 4)**

| **Chromosome** | **Location** | **No. of SNPs*** | **Gene** |
| --- | --- | --- | --- |
| 1 | 101851482 | 1 | RP11-157N3.1 (lincRNA) |
| 1 | 103755600 | 1 | Intergenic |
| 1 | 109070167 | 1 | Intergenic |
| 1 | 114630097-114675076 | 4 | *SYT6* |
| 1 | 116717522 | 1 | Intergenic |
| 1 | 151792842-151817543 | 2 | *RORC, C2CD4D, THEM5* |
| 1 | 161968072 | 1 | *OLFML2B* |
| 1 | 175850011 | 1 | Intergenic |
| 2 | 13134854 | 1 | AC064875.2 |
| 2 | 56002607-57936992 | 3 | *EFEMP1, CCDC85A*, AC007743.1 |
| 2 | 76944275 | 1 | Intergenic |
| 2 | 137173396-137872490 | 2 | *THSD7B* |
| 2 | 183699180-185281789 | 3 | *DUSP19, NUP35* |
| 2 | 202847242 | 1 | Intergenic |
| 2 | 207067503 | 1 | *GPR1* |
| 2 | 212380286-213576272 | 3 | *ERBB4* |
| 3 | 43794949 | 1 | Intergenic |
| 3 | 45606651 | 1 | *LIMD1* |
| 3 | 105695408 | 1 | Intergenic |
| 3 | 112913318 | 1 | Intergenic |
| 3 | 194538730 | 1 | Intergenic |
| 4 | 4275260 | 1 | *LYAR* |
| 4 | 99541944-99548762 | 2 | *TSPAN5* |
| 4 | 100334943 | 1 | *ADH7* |
| 4 | 107940588-107943491 | 2 | *DKK2* |
| 4 | 135796170 | 1 | Intergenic |
| 5 | 79086960 | 1 | *CMYA5* |
| 5 | 99269809 | 1 | Intergenic |
| 5 | 114127581 | 1 | Intergenic |
| 5 | 118671874 | 1 | *TNFAIP8* |
| 5 | 120965514 | 1 | Intergenic |
| 5 | 147289856 | 1 | Intergenic |
| 5 | 156626337 | 1 | *ITK* |
| 6 | 21233412 | 1 | *CDKAL1* |
| 6 | 25411435 | 1 | *LRRC16A* |
| 6 | 27247668-27396321 | 3 | *POM121L2, VN1R10P, ZNF204P, ZNF391, MCFD2P1* |
| 6 | 29937493-33853641 | 94 | Major Histocompatibility Complex |
| 6 | 35337931-35732137 | 9 | *PPARD, MKRNP2, FANCE, TEAD3, RPL10A, TULP1, FKBP5, ARMC12* |
| 6 | 72805811-72828559 | 3 | *RIMS1* |
| 6 | 106410424 | 1 | Intergenic |
| 6 | 111924913 | 1 | *TRAF3IP2* |
| 6 | 130512490-130537430 | 6 | *SAMD3* |
| 7 | 8240341-8243193 | 3 | *ICA1* |
| 7 | 20123972 | 1 | AC005062.2 |
| 7 | 22161810 | 1 | *RAPGEF5* |
| 7 | 89333692 | 1 | Intergenic |
| 7 | 141072134-141085654 | 3 | *TMEM178B* |
| 8 | 72534277 | 1 | Intergenic |
| 9 | 8710098 | 1 | *PTPRD* |
| 9 | 24423134 | 1 | Intergenic |
| 9 | 111621283 | 1 | Intergenic |
| 10 | 56913475 | 1 | *PCDH15* |
| 10 | 76833088 | 1 | Intergenic |
| 10 | 79178467 | 1 | *KCNMA1* |
| 10 | 94841988 | 3 | Intergenic |
| 11 | 15169639-15177816 | 3 | *INSC* |
| 11 | 73714650 | 1 | *UCP3* |
| 12 | 28214312-28237731 | 4 | Intergenic |
| 12 | 29659037 | 1 | *TMTC1* |
| 12 | 58840232 | 1 | Intergenic |
| 12 | 62396765 | 1 | *FAM19A2* |
| 12 | 70951978 | 1 | *PTPRB* |
| 12 | 79314798-79741443 | 3 | *SYT1* |
| 12 | 83061803-83101314 | 3 | *TMTC2* |
| 12 | 96544302 | 1 | Intergenic |
| 12 | 102331085 | 1 | *DRAM1* |
| 12 | 108703455 | 1 | *CMKLR1* |
| 13 | 48726060 | 3 | Intergenic |
| 13 | 69768976-69772154 | 1 | Intergenic |
| 14 | 81127849 | 1 | *CEP128* |
| 15 | 64185344 | 1 | Intergenic |
| 15 | 77282884-77296134 | 3 | *PSTPIP1* |
| 16 | 22943188 | 1 | Intergenic |
| 16 | 57009165 | 1 | *CETP* |
| 16 | 65902516 | 1 | Intergenic |
| 16 | 72916326-73133159 | 3 | *ZFHX3* |
| 16 | 85616985 | 1 | RP11-118F19.1 (lincRNA) |
| 17 | 3496105 | 1 | *SHPK, TRPV1* |
| 17 | 3498411- 3527281 | 2 | *SHPK, TRPV1* |
| 17 | 3632836-3689132 | 3 | *ITGAE* |
| 17 | 45316717 | 1 | Intergenic |
| 18 | 51448760 | 1 | Intergenic |
| 19 | 38743962-38900106 | 14 | *PPP1R14A, SPINT2, C19orf33, YIF1B, KCNK6, CATSPERG, PSMD8, SPRED3, GGN, FAM98C* |
| 20 | 47403913-47420680 | 3 | *PREX1* |

* number of SNPs with an absolute iHS score greater than four.
