# Supplementary material for: Novel genetic polymorphisms associated with severe malaria and under selective pressure in North-eastern Tanzania
Source: PLoS Genet. 2018 Jan 30;14(1):e1007172. doi: 10.1371/journal.pgen.1007172 (PMC5806895; doi:10.1371/journal.pgen.1007172)
Supplement: S5 Table — (DOCX) [file pgen.1007172.s006.docx]

**S5 Table: Regions under potential significant differential selection between cases and controls (XP-EHH > 4)**.

| **Chromosome** | **Position** | **No. SNPs*** | **Gene** |
| --- | --- | --- | --- |
| 6 | 13824087 | 1 | *MCUR1* |
| 6 | 31066671 | 1 | Major Histocompatibility Complex |
| 6 | 53351289 | 1 | *GCLC* |
| 7 | 53105223 | 1 | *POM121L12* |
| 14 | 70875513-71047754 | 5 | *SYNJ2BP*, *ADAM21*, *ADAM20* |
| 21 | 40093658 | 1 | *ERG, ETS2* |

* Number of SNPs with an absolute XP-EHH score greater than four.
